# English as a foreign language writing anxiety and its relationship with self-esteem and mobile phone addiction among Chinese medical students—A structural equation model analysis

**DOI:** 10.1371/journal.pone.0284335

**Published:** 2023-04-20

**Authors:** Yang Song, Kristin Sznajder, Qiuye Bai, Yanyan Xu, Yifei Dong, Xiaoshi Yang

**Affiliations:** 1 Department of Applied Linguistics, Institute of Foreign Languages, China Medical University, Shenyang, Liaoning, P. R. China; 2 Department of Public Health Sciences, College of Medicine, Pennsylvania State University, Hershey, Pennsylvania, United States of America; 3 Department of Stomatology, Benxi Central Hospital, Benxi, Liaoning, P. R. China; 4 Department of Clinical Oncology, Shengjing Hospital of China Medical University, Shenyang, Liaoning, P. R. China; 5 Department of Physics, Liaoning Normal University, Dalian, Liaoning, P. R. China; 6 Department of Social Medicine, College of Health Management, China Medical University, Shenyang, Liaoning, P. R. China; Ahvaz Jundishapur University: Ahvaz Jondishapour University of Medical Sciences, ISLAMIC REPUBLIC OF IRAN

## Abstract

Medical students in China and other non-English speaking countries are susceptible to English as a Foreign Language (EFL) writing anxiety. English writing is not only a vital component tested for admission to postgraduate and doctoral programs, but it is also critical for the publication of academic papers. Although there is mounting evidence indicating relationships between anxiety, self-esteem and mobile phone addiction, pathways between these three constructs within a structural equation model have not yet been examined. Furthermore, there has been a dearth of studies exploring EFL writing anxiety, from which medical students in China as well as other non-English speaking countries are prone to suffer. The study was to assess EFL writing anxiety among Chinese medical students and to examine the relationships between EFL writing anxiety, self-esteem, and mobile phone addiction, with an aim to offer empirical evidence for effective preventive or intervention measures to alleviate EFL writing anxiety. Cross-sectional data were obtained from 1,238 medical students in China, with the administration of a self-administered questionnaire comprising the Second Language Writing Anxiety Inventory (SLWAI), the Rosenberg Self Esteem Scale (RSES) and the Mobile Phone Addiction Tendency Scale (MPATS). The results indicated that both self-esteem and mobile phone addiction exerted significant direct effects on EFL writing anxiety. Self-esteem also had a significant indirect effect on EFL writing anxiety via the mediating role of mobile phone addiction. The path coefficients of self-esteem on EFL writing anxiety were significantly reduced when mobile phone addiction was modeled as a mediator. Efforts to alleviate EFL writing anxiety among medical students may benefit from enhancing self-esteem and establishing a healthy relationship with mobile phones.

## Introduction

English as a Foreign Language (EFL) learning has been the focus of a wealth of research efforts due to the accepted role of English as a lingua franca in academic settings and numerous other domains of globalization [1,2]. Mounting evidence has identified anxiety as one of the primary sources of barriers that EFL learners encounter during language learning process [3–5]. Anxiety associated with EFL writing has begun to draw growing attention due to its widespread existence and influence among EFL learners [6]. Chinese students have devoted constant concern to EFL learning, since better command of English (mostly represented by higher English test scores) can help them gain a competitive edge throughout both educational and job-hunting periods [7,8]. EFL writing has been placed emphasis on for its consistently being included as an indispensable part of the English test of China’s College Entrance Exam, the national College English Test Band 4 (CET-4) and Band 6 (CET-6) (the passing score of which are a necessity either to obtain an undergraduate diploma, or to enter postgraduate programs or employment) [7]. With regards to medical students, EFL writing could be perceived as of more significance, for English writing competence is not only inevitably tested in the examinations for postgraduate and doctoral admission but also plays a vital role for the publication of articles listed in the Science Citation Index, which has been widely considered as a crucial assessment factor in the existing appraisal system for the medical profession in China [9]. Chinese medical students could therefore be more likely to be susceptible to EFL writing anxiety. Despite increasing concern in other non-English speaking countries [10], EFL writing anxiety among medical students in China remains less reported. Meanwhile, although there is mounting evidence indicating relationships between anxiety, self-esteem and mobile phone addiction among university students, the pathways between these three constructs within a structural equation model has not been reported and no literature has explored the mediating role mobile phone addiction may exhibit in the relationship between self-esteem and anxiety in the language learning contexts. Thus, the current study concentrated on the association between EFL writing anxiety, self-esteem and mobile phone addiction among Chinese medical students and went deeper by examining how mobile phone addiction could act as a mediator, attempting to provide more empirical evidence for developing effective interventions to alleviate EFL writing anxiety.

### EFL writing anxiety

Foreign language writing involves not only the grammatical and lexical accuracy but cultural appropriateness to construct content [11,12], which is considered to be demanding and challenging for language learners [13]. Foreign language writing anxiety, categorized as a situation-specific anxiety occurring in the language learning contexts, refers to the experience of feelings of tension and apprehension that interfere with the writing process [13,14]. There is accumulated evidence that debilitating anxiety associated with writing can negatively influence English as a Foreign Language (EFL) writing motivation and writing performance, since it can cause feelings of anguish and detestation towards writing and difficulty in producing effective and logical writing [15]. EFL learners with higher levels of writing anxiety were reported to write shorter and less qualified compositions compared with their less anxious counterparts [13,16]. Given the widespread existence and well-documented impacts of writing anxiety in the EFL learning context, mounting research has been dedicated to determining the levels and types of EFL writing anxiety and to identifying the individual and contextual factors that contribute to the increased likelihood of EFL writing anxiety [16–22].

### Self-esteem

Self-esteem, as one of learners’ individual affective factors, is conceptualized as an individual’s personal assessment of self-worth [23], which signifies the evaluative dimension of self-concept and portrays how individuals feel about themselves [24]. Self-esteem has been found to function as a cushion against negative emotions, to enhance coping, and to contribute positively to physical and mental health [25–27]. Furthermore, a growing body of research has revealed the alleviating effects of self-esteem on anxiety [28,29]. Cross-sectional studies conducted among adolescents, young adults and university students have echoed that those with lower self-esteem were found to be more likely to experience higher levels of anxiety compared with those who have higher self-esteem [28,30,31]. There has also been longitudinal research confirming the predictive role of low self-esteem on anxiety [29]. Language researchers have recently begun to focus more attention on the role that self-esteem plays in foreign language learning [32]. Self-esteem has been revealed to enhance language self-confidence, aspiration, and adjustment to perception of foreign language structure among language learners [33]. It has also been documented to reliably predict language learners’ achievement [34]. With respect to EFL writing, a significant negative association has been found to exist between writing anxiety and self-esteem. University students with low self-esteem tend to feel more anxious about EFL writing than their high self-esteem counterparts [35]. Compared with the extensive body of literature pertaining to the relationship between anxiety and self-esteem, empirical evidence exploring that in the EFL writing context is still limited.

### Mobile phone addiction

Mobile phone addiction has emerged as an increasingly serious issue which has aroused wide public concern. Admittedly, the convenient internet connectivity and user-friendly features of mobile phones enable them to be the most widely-used internet-access device [36] and to play an irreplaceable role in both communication and education for students [37]. Nonetheless, research has increasingly recognized the risk of mobile phone addiction could be raised due to prolonged and excessive use of mobile phones among university students, which in turn may lead to higher anxiety levels and eventually could hinder their academic growth and overall well-being [37–39]. Although the definite diagnostic criteria of mobile phone addiction are yet to be established and consensus on the frequency and duration by which mobile phone use can be determined as problematic is yet to be achieved, mobile phone addiction has generally been recognized as an addictive disorder exhibiting one’s excessive or uncontrolled use of mobile phones which leads to physical, psychological, and social impairments [40,41]. Mobile phone addiction among medical students is becoming a growing concern, with recent studies revealing that the prevalence of mobile phone addiction among medical students was as high as 36.8% in Nepal [37] and 29.8% in China [39]. Self-esteem has been found to be one of the positive psychological variables that could reduce the risk of mobile phone addiction [42–44]. Meanwhile, empirical evidence has emerged revealing the significant effect of mobile phone addiction on anxiety. Mobile phone addiction was shown to be positively associated with anxiety among university students [45,46]. A recent systematic review and meta-analysis also confirmed the finding that college students with mobile phone addiction tended to have high levels of anxiety [47]. Based on findings from previous research, it can be assumed that mobile phone addiction might mediate the relationship between self-esteem and anxiety.

### The current study

With findings from prior research raising the possibility that relationships might exist between anxiety, self-esteem and mobile phone addiction among university students, the pathways between these three constructs within a structural equation model has not yet been examined. Furthermore, despite mounting evidence linking either self-esteem or mobile phone addiction with anxiety, there has been a dearth of studies exploring EFL writing anxiety in the language learning contexts, with which medical students in China as well as other non-English speaking countries are prone to experience. Therefore, the study was to assess EFL writing anxiety among Chinese medical students and to examine the relationships between EFL writing anxiety, self-esteem and mobile phone addiction, with an aim to offer empirical evidence for effective preventive or intervention measures to alleviate EFL writing anxiety. Two hypotheses formulated for the present study were thus tested: (1) self-esteem and mobile phone addiction are positively associated with writing anxiety and (2) mobile phone addiction plays a mediating role in the relationship between self-esteem and writing anxiety among medical students in China.

## Methods

### Study design and participants

This study was conducted online from March to April in 2021 in China Medical University and Dalian Medical University, located in Liaoning province in the northeast of China. The study incorporated a cross-sectional survey design using stratified sampling methods. The sample consists of first-year, second-year and third-year medical students from 24 randomly-selected classes (with 8 classes from each grade) at China Medical University and Dalian Medical University. Eligibility for study participation included being 18 years old or older, able to read and respond in Chinese, and have access to the internet. A self-administered electronic questionnaire pertaining to demographic characteristics, EFL writing anxiety, self-esteem and mobile phone addiction was distributed via a widely-recognized professional online survey platform in China (“Wenjuanxing”) and the questionnaire link was sent to participants through the most widely-used social media applications (WeChat and Tencent QQ). Participants clicked the link to start filling the questionnaire and clicked the submit button to complete the submission once they finish it. The questionnaire was configured to ensure that it could be submitted only when all the questions had been fully completed and participants could go back to the previous questions and revise them before submitting their responses, which prevented the possibility of incomplete, inconsistent and duplicate data. After the elimination of the questionnaires with illogical answers, a logic and data consistency test was conducted, the result of which fulfilled the prerequisites of statistical analysis. The questionnaire was completely anonymous and confidential, which took approximately 15 to 20 minutes to finish. A total of 1,238 medical students provided complete and valid responses, thus constituting our final sample with the valid response rate being 88.7%.

### Ethics statement

This study was conducted in accordance with the Helsinki declaration and was approved by the Committee on Human Experimentation of China Medical University. All participants were fully informed of the purpose and contents of study and provided written informed consent before participation.

### Measures

#### Demographic characteristics

Demographic information collected in this study included age, gender, year of medical education, major, family monthly income, parents’ level of education, family location and history of major diseases (e.g. cardiovascular diseases, diabetes, virulent tumors and respiratory diseases).

#### EFL anxiety

EFL writing anxiety was assessed based on the Second Language Writing Anxiety Inventory (SLWAI), which was developed by Cheng (2004) as a reliable measurement instrument for learners’ anxiety experiences during second or foreign language writing [16]. SLWAI consists of three sub-scales which include 22 Likert-type items conforming to a multidimensional conceptualization of anxiety [48]. The three subscales of SLWAI measure Somatic anxiety (“as reflected in negative feelings”), Cognitive anxiety (“as reflected in negative expectations, or worry or fear of negative evaluation”), and Avoidance behaviour (“as reflected in avoidance in writing”), respectively [16]. We used 18 items of SLWAI which counted towards the total score of EFL writing anxiety in this study. The Cronbach’s alpha of SLWAI in this study was 0.886, which is considered to demonstrate good reliability.

#### Self-esteem

Self-esteem was measured with the Rosenberg Self Esteem Scale (RSES), which has been widely used and adapted into different languages with adequate reliability and validity [49]. It consists of 10 four-point Likert items measuring both the positive and the negative self-evaluation. Items 1, 2, 4, 6, and 7 are positively rated and items 3, 5, 8, 9, and 10 are negatively rated, with a total score ranging from 0 to 30 and higher scores indicative of higher levels of self-esteem [23]. The Cronbach’s alpha of RSES of this study was 0.863, showing good reliability.

#### Mobile phone addiction

Mobile phone addiction was assessed with the Mobile Phone Addiction Tendency Scale (MPATS), the most widely used tool for measuring mobile phone addiction among Chinese college students [50]. It comprises 16 four-point Likert items assessing 4 domains of mobile phone addiction tendency, including perceptions of withdrawal symptoms, salience, social comfort and mood changes, with higher total scores indicative of a greater tendency of mobile phone addiction [51]. The Cronbach’s alpha of MPATS of present study was 0.942, indicating good reliability.

### Statistical analyses

SPSS 17.0 was used for the statistical analyses in this study with statistical significance determined by a two-tailed probability value of <0.05. T-tests and one-way ANOVA were conducted to test significant differences in EFL writing anxiety among the categorical variables. Descriptive statistics were calculated to summarize the distribution of demographic characteristics, self-esteem, mobile phone addiction and EFL writing anxiety. Correlations between self-esteem, mobile phone addiction, and EFL writing anxiety were assessed with Spearman’s correlation. Hierarchical regression analysis (HMR) was employed to measure the effects of demographic characteristics, self-esteem and mobile phone addiction on EFL writing anxiety. Blocks of independent variables including demographic characteristics, self-esteem and mobile phone addiction were entered in the regression model successively so that their incremental contributions to the HMR model could be examined.

Structural Equation Model (SEM) analysis was conducted by using Amos 17.0 to test whether mobile phone addiction played a mediating role between self-esteem and EFL writing anxiety. EFL writing anxiety and self-esteem were modeled as the dependent variable and the independent variable, respectively, with mobile phone addiction as the mediator. All indices involved were fitted with the SEM criteria (χ^2^/df < 5, RMSEA < 0.08, GFI > 0.90, CFI > 0.90, and TLI > 0.90), demonstrating good fitness. When mobile phone addiction was added, decrease of the direct path coefficient of self-esteem on EFL writing anxiety would indicate a mediating effect of mobile phone addiction. The bootstrapping procedure based on 5000 bootstrapping sample, a bias-corrected and accelerated 95% confidence interval (BCa 95% CI), and 95% percentile confidence intervals (percentile 95% CI) was adopted in this study, with a two-tailed probability value of <0.05 being considered statistically significant.

## Results

### Description of the participants

Demographic characteristics of the participating medical students are illustrated in Table 1. The average age of the 1,238 participants was 19.40 (SD ±1.10), ranging from 18 to 26 years old. Approximately two-thirds (65.99%) were females. Nearly half of the participants were first-year medical students (48.47%) and 51.53% of the sample belonged to second-year or higher levels of medical education. More than two-fifths (43.30%) majored in clinical medicine, while 56.70% of the sample enrolled in other medical science majors. Approximately two-fifths (39.90%) came from families with a monthly income above 6000 yuan RMB. The majority of the participants (92.73%) reported no significant past medical history.

**Table 1:** Demographic characteristics and distributions of EFL writing anxiety among college students.

Variables	N (%)	Writing Anxiety (Mean ±SD)	Somatic anxiety (Mean ±SD)	Cognitive anxiety (Mean ±SD)	Avoidance anxiety (Mean ±SD)
**Age (yr)**					
≤19	705(56.95)	53.35±11.05	17.48±5.00	17.35±3.99	18.52±3.82
>19	533(43.05)	54.80±10.93*	18.08±4.73*	17.83±3.90*	18.89±3.91
**Gender**					
Male	421(34.01)	54.04±11.25	18.03±5.15	17.26±4.06	18.75±3.81
Female	817(65.99)	53.94±10.91	17.59±4.76	17.71±3.90	18.63±3.89
**Grade**					
1	600(48.47)	54.07±10.81	17.77±4.95	17.64±3.88	18.72±3.70
≥2	638(51.53)	53.89±11.22	17.71±4.85	17.48±4.03	18.63±4.01
**Major**					
Clinical medicine	536(43.3)	53.93±11.66	17.65±5.17	17.55±4.21	18.74±4.10
Other	702(56.7)	54.01±10.52	17.81±4.68	17.56±3.75	18.63±3.67
**Family monthly income**					
<3000	249(20.11)	55.57±9.80**	18.25±4.49**	18.22±3.59**	19.09±3.38
3001–6000	495(39.98)	54.43±10.60**	17.97±4.68**	17.70±3.75*	18.75±3.72
>6000	494(39.90)	52.71±11.87	17.25±5.25	17.07±4.27	18.39±4.20
**Father’s Education**					
High school and below	757(61.15)	54.90±10.42**	18.15±4.60**	17.87±3.78**	18.89±3.75*
College and above	481(38.85)	52.52±11.77	17.10±5.27	17.06±4.18	18.35±4.01
**Mother’s Education**					
High school and below	800(64.62)	54.99±10.26**	18.14±4.63**	17.90±3.72**	18.95±3.67**
College and above	438(35.38)	52.13±12.08	17.01±5.28	16.92±4.30	18.19±4.15
**Family Location**					
Urban area	671(54.2)	53.01±11.65	17.35±5.16	17.23±4.16	18.43±4.09
Rural area	567(45.8)	55.11±10.13**	18.20±4.52**	17.95±3.67**	18.97±3.56*
**Medical history**					
Yes	90(7.27)	55.35±11.11	17.94±5.02	18.07±4.03	19.34±4.41
No	1148(92.73)	53.87±11.01	17.72±4.89	17.52±3.95	18.63±3.81

Note:

*P<0.05,

**P<0.01.

Abbreviations: EFL, English as a foreign language.

Medical students aged over 19 years old reported a higher level of EFL writing anxiety than those aged 19 or below (*P*<0.05). Those whose family monthly income was over 6000 yuan RMB tended to be less anxious about EFL writing than those whose family monthly income was below that level (*P*<0.01). Those whose parents received college education or above had lower scores of EFL writing anxiety than those whose parents have not attended college (*P*<0.01). Medical students from rural areas were more susceptible to EFL writing anxiety than those from urban areas (*P*<0.01).

### Correlations between EFL writing anxiety, self-esteem and mobile phone addiction

As illustrated in Table 2, both self-esteem and mobile phone addiction were significantly associated with EFL writing anxiety (*P*<0.01). Specifically, self-esteem was significantly negatively correlated with EFL writing anxiety (*P*<0.01), while mobile phone addiction was significantly positively correlated with EFL writing anxiety (*P*<0.01).

**Table 2:** Correlations among EFL writing anxiety, self-esteem, and mobile phone addiction.

**Variables**	**M**	**SD**	**1**	**2**	**3**	**4**
**1.EFL writing anxiety**	53.98	11.02	1			
**2.Age**	19.40	1.10	0.045	1		
**3.Self-esteem**	29.92	4.79	-0.353**	-0.032	1	
**4.Mobile phone addiction**	45.96	11.91	0.475**	0.047	-0.336**	1

Note:

**P<0.01.

Abbreviations: EFL, English as a foreign language.

### The hierarchical linear regression analysis of EFL writing anxiety

As shown in Table 3, the final HMR model contributed to a total of 28.30% of the variance in EFL writing anxiety. Self-esteem showed a significant and negative association with EFL writing anxiety, explaining 11.70% of the total variance. Mobile phone addiction exhibited a significant and positive association with EFL writing anxiety, accounting for 14.30% of the total variance. Results of this study also revealed that the effect of self-esteem on EFL writing anxiety among medical students might be partially mediated by mobile phone addiction. The regression coefficient (β) for the association between self-esteem and EFL writing anxiety was decreased from -0.351 to -0.215 when mobile phone addiction was entered into the model.

**Table 3:** The hierarchical multiple regression analysis of EFL writing anxiety.

Variable	Model 1			Model 2			Model 3		
	β	Standardized β	95% CI	β	Standardized β	95% CI	β	Standardized β	95%CI
***Block 1*. *Demographic characteristics***									
**Age (years)**	0.031	0.031	-0.036–0.099	0.032	0.032	-0.031–0.095	0.031	0.031	-0.027–0.089
**Gender** (male vs. female)	0.006	0.003	-0.113–0.125	-0.1	-0.048	-0.213–0.013	-0.106*	-0.050*	-0.209--0.003
**Grade** (first year vs. other)	0.04	0.02	-0.099–0.179	0.045	0.023	-0.085–0.176	0.096	0.048	-0.023–0.216
**Major** (clinical medicine vs. other)	0.049	0.024	-0.071–0.17	0.088	0.043	-0.026–0.201	0.113	0.056	0.009–0.216
**Family monthly income** (<¥3000 vs. >¥6000)	0.127	0.051	-0.044–0.298	0.094	0.038	-0.066–0.254	0.056	0.022	-0.091–0.202
**Family monthly income** (¥3001–6000 vs. >¥6000)	0.08	0.039	-0.053–0.213	0.091	0.045	-0.033–0.216	0.086	0.042	-0.028–0.2
**Father’s education level** (high school and below vs. college and above)	0.062	0.03	-0.088–0.212	0.025	0.012	-0.116–0.166	0.047	0.023	-0.082–0.176
**Mother’s education level** (high school and below vs. college and above)	0.143	0.068	-0.013–0.299	0.1	0.048	-0.046–0.247	0.08	0.038	-0.054–0.213
**Family location** (urban area vs. rural area)	-0.071	-0.035	-0.197–0.055	-0.073	-0.036	-0.191–0.045	-0.07	-0.035	-0.178–0.038
**Medical history** (yes vs. no)	0.108	0.028	-0.106–0.323	-0.039	-0.01	-0.242–0.163	0.037	0.01	-0.148–0.223
***Block 2*. *Self esteem***				-0.351**	-0.351**	-0.404--0.298	-0.215**	-0.215**	-0.267--0.164
***Block 3*. *Mobile phone addiction***							0.403**	0.402**	0.353–0.454
**R** ^**2**^		**0.023**			**0.140**			**0.283**	
**Adjusted R** ^**2**^		**0.015**			**0.132**			**0.276**	
**ΔR** ^**2**^		**0.023**			**0.117**			**0.143**	

Note:

*P<0.05,

**P<0.01.

Abbreviations: EFL, English as a foreign language.

### Structural equation modeling of the mediating effect of mobile phone addiction between self-esteem and EFL writing anxiety

**Fig 1:**
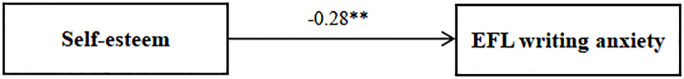
Standardized solutions for the structural equation model of self-esteem and EFL writing anxiety. Note: **P<0.01. The standardized path coefficient is illustrated on the unidirectional arrow path. **The coefficient of the path is significant at the P<0.01 level. Abbreviations: EFL, English as a foreign language.

**Fig 2:**
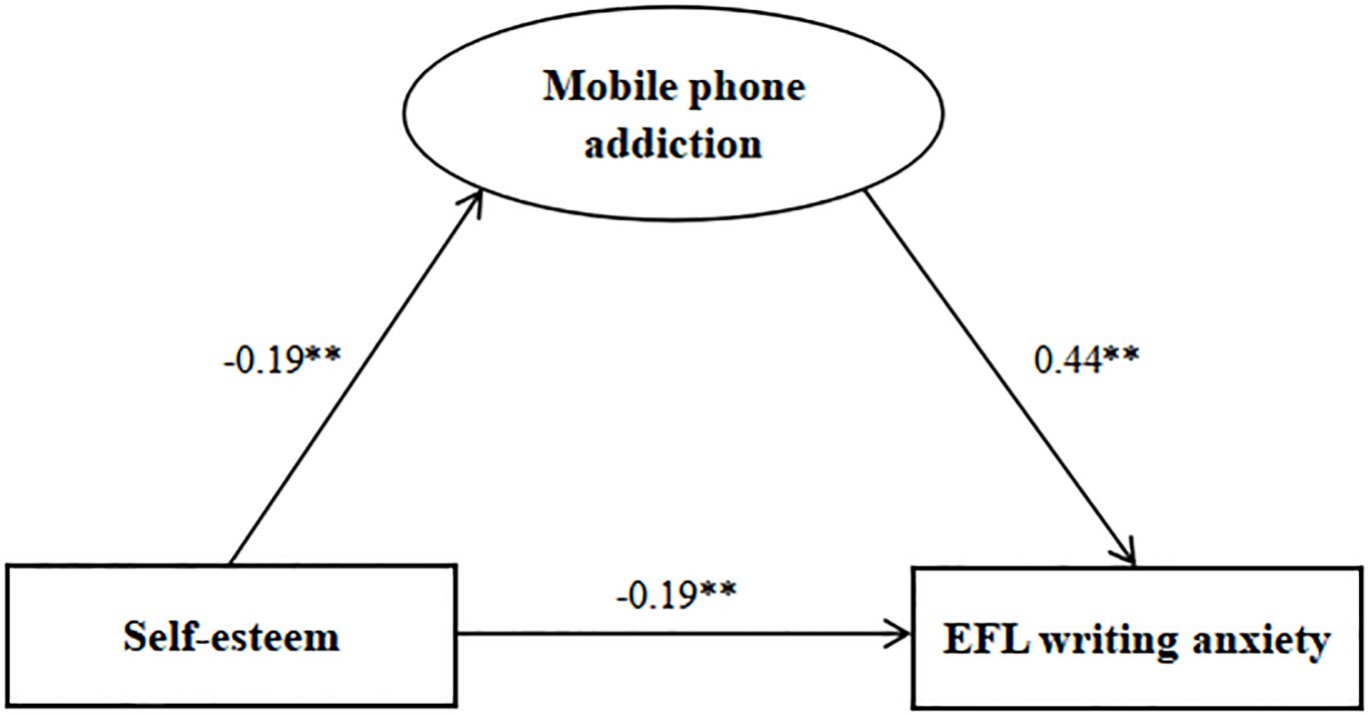
Standardized solutions for the structural equation model of the mediating effect of mobile phone addiction between self-esteem and EFL writing anxiety. Note: **P<0.01. Standardized path coefficients are illustrated on the unidirectional arrow paths. **The coefficient of the path is significant at the P<0.01 level. Abbreviations: EFL, English as a foreign language.

Table 4 illustrates the path coefficients for the SEM analysis, while Figs 1 and 2 present the standardized solutions for the SEM. As revealed in Fig 1, the direct effect of self-esteem on EFL writing anxiety was estimated (the model fit of the data χ^2^/df = 3.222<5, GFI = 0.982>0.90, AGFI = 0.965>0.90, CFI = 0.990>0.90, TLI = 0.983>0.90, RMSEA = 0.042<0.05), indicating that self-esteem had a significant direct effect on EFL writing anxiety (c = -0.28, P<0.01). Fig 2 shows a statistically significant effect of mobile phone addiction on both self-esteem (β = -0.19, *P*<0.01) and EFL writing anxiety (β = 0.45, *P*<0.01), with a high-level of goodness of fit (χ^2^/df = 3.810<5, GFI = 0.972>0.90, AGFI = 0.948>0.90, CFI = 0.984>0.90, TLI = 0.973>0.90, RMSEA = 0.048<0.05). The path coefficient of self-esteem on EFL writing anxiety was significantly decreased (β = -0.19, *P*<0.01) when mobile phone addiction was modeled as a mediating variable. It was revealed that mobile phone addiction played a statistically significant mediating role between self-esteem and EFL writing anxiety (a*b = -0.09, BCa 95% CI: -0.125 to -0.043, Percentile 95% CI: -0.125 to -0.042). Hence, self-esteem not only exerted a significant direct effect on EFL writing anxiety, but also had a significant indirectly effect on EFL writing anxiety via the mediating role of mobile phone addiction.

**Table 4:** The path coefficients of the structural equation model.

	B	β	S.E.	C.R.	P
Mobile phone addiction ← Self-esteem	-1.422	-0.19	0.251	-5.665	<0.001
EFL writing anxiety ← Mobile phone addiction	0.723	0.44	0.068	10.614	<0.001
EFL writing anxiety ← Self-esteem	-2.383	-0.19	0.509	-4.683	<0.001

Abbreviations: EFL, English as a foreign language; B, the unstandardized path coefficient; β, the standardized path coefficient; S.E., the standard error; C.R., the critical ratio; P, the significance level.

## Discussion

This study examined the mediating effect of mobile phone addiction in the relationship between self-esteem and EFL writing anxiety, which has not yet been explored in the existing literature. The finding of this study were congruent with previous studies confirming the alleviating effect of self-esteem on anxiety and went further by proving its negative effect on EFL writing anxiety. This study echoed findings from recent cross-sectional studies conducted among university students in Spain, Vietnam and Indian, respectively, confirming that those with lower self-esteem tended to exhibit higher levels of anxiety [28,30,31]. The finding of this study was also in line with a recent study conducted among high school students, which revealed the negative effect of self-esteem on text anxiety and math anxiety [52]. Moreover, this result coincided with the conclusion of a meta-analysis of 18 longitudinal studies on the association between self-esteem and anxiety indicating the prospective role of self-esteem in predicting anxiety [29]. The EFL writing process is a demanding and stressful experience that requires the application and integration of multiple abilities and skills [19]. The existence of anxiety during EFL writing has been found to be caused mainly by linguistic difficulties and the fear of negative evaluation [10,19]. The protective effect of self-esteem against EFL writing anxiety may be attributed to the association between self-esteem and psychological adjustment suggested by the Terror Management Theory [53] and the vulnerability model [54]. Those with high self-esteem tend to employ their positive resources interchangeably when exposed to a threat to their self-worth, whereas those with low self-esteem are less likely to possess a rich reservoir of positive views pertaining to their self-concept, which makes them more vulnerable when their self-worth is threatened [55]. Medical students with higher self-esteem might be more likely to resort to their positive resources and be less fragile when facing challenges and negative evaluation during EFL writing process, thus being less susceptible to EFL writing anxiety.

This study extended the existing research by revealing that self-esteem not only exerts a direct effect on EFL writing anxiety but also has an indirect effect on it through the mediating path of mobile phone addiction. This negative influence of self-esteem on mobile phone addiction revealed in this study lends support to previous studies conducted among college students which indicated that self-esteem could be an important predictor for mobile phone addiction [42,56]. One possible explanation is that those with low self-esteem are more inclined to seek security in affective relationships [44], which usually results in excessive mobile phone use to satisfy their psychological needs [57].

Meanwhile, this study revealed that mobile phone addiction was positively associated with EFL writing anxiety among medical students, which was to some extent congruent with prior research indicating that mobile phone addiction was linked to an increased likelihood of higher levels of anxiety [45,46]. A recent preliminary cross-sectional study conducted among Bulgarian and foreign medical students found that those addicted to mobile phone tended to experience higher levels of anxiety [45]. Another study using structural equation modelling conducted among Lebanese undergraduate students also revealed mobile phone addiction was indicative of a greater risk of higher levels of anxiety [46]. Results from this study also echoed findings of a recent systematic review and meta-analysis which identified 40 studies involving 33,650 college students altogether indicating that mobile phone addiction could be a positive predictor of anxiety [47]. Outcomes of mobile phone addiction could bear a close resemblance to those of internet addiction in terms of negative emotions, since functions of mobile phones enjoy similar features of computers and the internet [58]. One possible reason for the link of mobile phone addiction to higher levels of anxiety might be that students addicted to mobile phones are prone to experience face-to-face social isolation and the shrinking of real-world social networking, which could raise the risk of frustrating personal companionship and reducing social support resources, subsequently resulting in higher levels of anxiety [58]. Admittedly, according to the reciprocal interplay between behaviors and emotions proposed by the social cognitive theory [59], individuals’ behaviors (e.g. mobile phone addiction) may not only affect their emotions but also be affected by their emotions (e.g. anxiety). There is also likelihood that mobile phone addicts resort to mobile phones aiming to cope with or reduce their anxiety but their maladaptive or avoidant coping could aggravate rather than ease their anxiety [58]. Hence, medical students with low self-esteem may be at a higher risk of mobile phone addiction due to their excessive security seeking in affective relationships or avoidance of negative self-perception by means of self-destructive behaviors. Furthermore, mobile phone addiction among medical students, a number of whom might rely on mobile phones to cope with or alleviate their EFL writing anxiety, might subsequently shrink their face-to-face interaction and real-world social networking with their peers and teachers who could contribute to reducing their language learning anxiety [60,61] and provide guidance and support with EFL writing [13,62,63]. Therefore, reduced social support might subsequently contribute to elevated EFL writing anxiety.

Meanwhile, findings of this study indicated that increased age, lower family income, lower education background of parents, and rural hometowns are linked to higher levels of EFL anxiety. One possible interpretation for these findings is that older medical students may be more aware of the importance of English writing competence both for medical education and for their future career as medical professionals. Meanwhile, those whose parents have comparatively lower levels of education and those who come from rural hometowns might have fewer social support resources available to them to improve English writing, which could also exacerbate their EFL writing anxiety.

Findings of this study have potential implications for the design of foreign language teaching programs and curriculum that may aid in alleviating writing anxiety and facilitate language learning for EFL learners. This study extends existing literature by providing empirical evidence on the mediating role of mobile phone addiction in the association between self-esteem and EFL writing anxiety among Chinese medical students. It appeals for more attention to EFL writing anxiety among both language researchers and teachers and highlights the importance of better preparing medical students for addressing EFL writing anxiety. EFL medical students could be offered more opportunity to obtain insight into the underlying mechanism of the effects of self-esteem and mobile phone addiction on EFL writing anxiety. Also, more emphasis could be placed on individual affective factors in the pedagogical practices of EFL writing instruction and activities aimed at alleviating EFL writing anxiety could devote more efforts to enhancing self-esteem among EFL learners. Furthermore, EFL learners should be informed of the detrimental impact of smartphone addiction and provided with training programs to establish a healthy relationship with mobile phones and effectively interact with teachers or peers so that they could be better equipped to fulfill foreign language writing demands with lower levels of anxiety.

One major limitation of this study is its cross-sectional design, in which no causal relationships between self-esteem, mobile phone addiction, and EFL writing anxiety could be concluded. Bidirectional relationships between these three variables might exist. Prospective studies are therefore necessary to provide longitudinal evidence for causal directions between self-esteem, mobile phone addiction, and EFL writing anxiety. Second, despite the large sample size and high response rate, participants were recruited from two medical universities located in two cities in one province of northeastern China, so the generalizability of the findings to a broader population of medical students needs to be verified by further empirical evidence.

## Conclusions

This study revealed that self-esteem exerts not only a direct effect but also an indirect effect on EFL writing anxiety through the mediating path of mobile phone addiction. Previous prevention and treatment of EFL writing anxiety mostly focus on students’ English competence without taking into consideration that students might be experiencing low self-esteem or mobile phone addiction. Hence, medical students in non-English speaking countries should be provided more opportunity to gain insight into the underlying mechanism of EFL writing anxiety and how self-esteem and mobile phone addiction could exert effects on EFL anxiety. Also, it is advisable that training programs on how to enhance self-esteem and establish a healthy relationship with mobile phones should be incorporated into the preclinical curriculum of medical education. Meanwhile, more activities involving interaction with teachers or peers could be taken into consideration to cope with challenges and alleviate anxiety in the EFL writing context.

## Supporting information

S1 Fig Standardized solutions for the structural equation model of self-esteem and EFL writing anxiety. (TIF)

S2 Fig Standardized solutions for the structural equation model of the mediating effect of mobile phone addiction between self-esteem and EFL writing anxiety. (TIF)

S1 Table Demographic characteristics and distributions of EFL writing anxiety among college students. (TIF)

S2 Table Correlations among EFL writing anxiety, self-esteem, and mobile phone addiction. (TIF)

S3 Table The hierarchical multiple regression analysis of EFL writing anxiety. (TIF)

S4 Table The path coefficients of the structural equation model. (TIF)

S1 Dataset The S1 Dataset includes the data concerning the study variables of the subjects in the present study. (XLSX)
