# Supplementary figures and images for: English as a foreign language writing anxiety and its relationship with self-esteem and mobile phone addiction among Chinese medical students—A structural equation model analysis
Source: PLoS One. 2023 Apr 20;18(4):e0284335. doi: 10.1371/journal.pone.0284335 (PMC10118181; doi:10.1371/journal.pone.0284335)

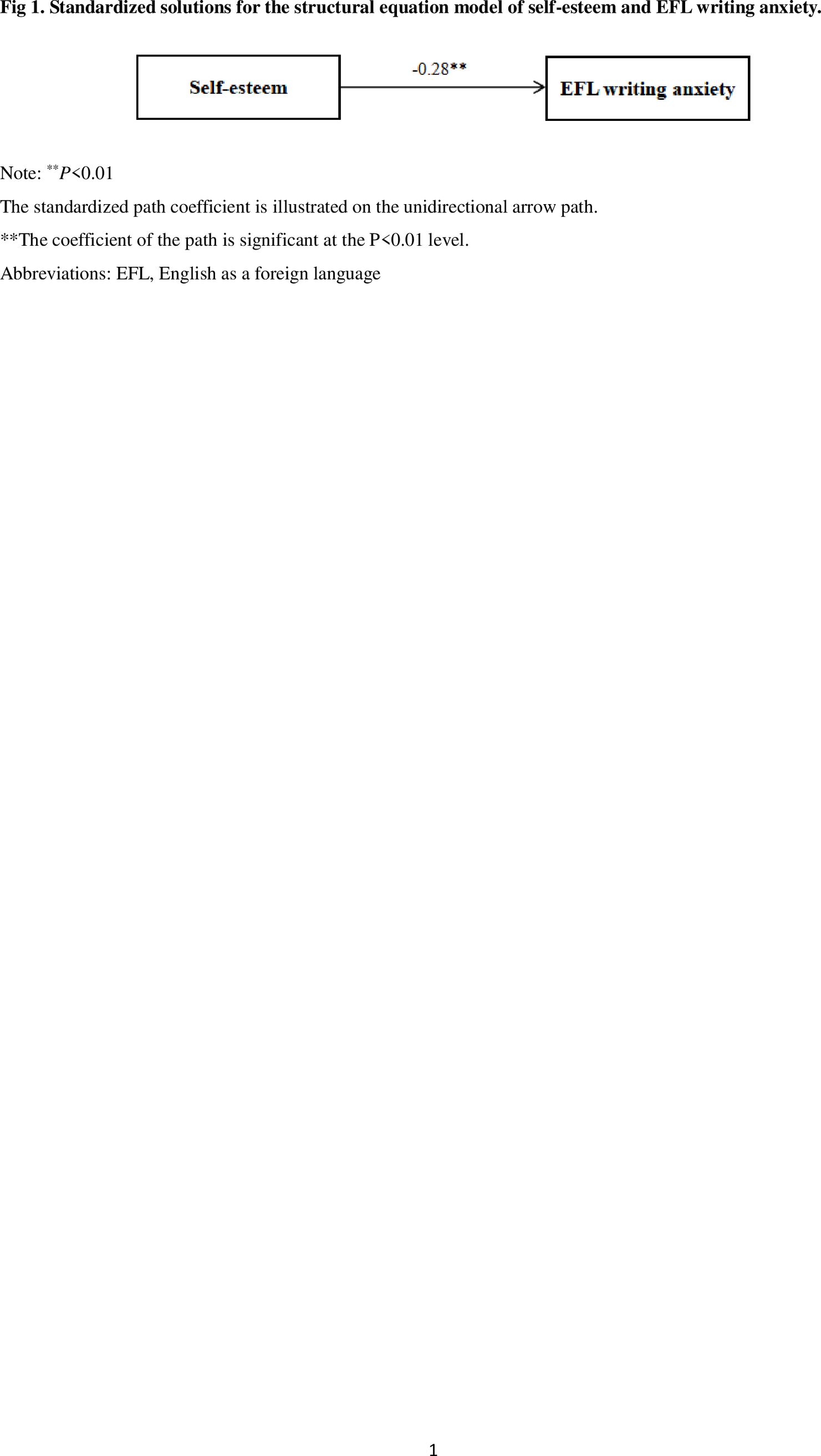

Supplement: S1 Fig — (TIF) [file pone.0284335.s001.tif]

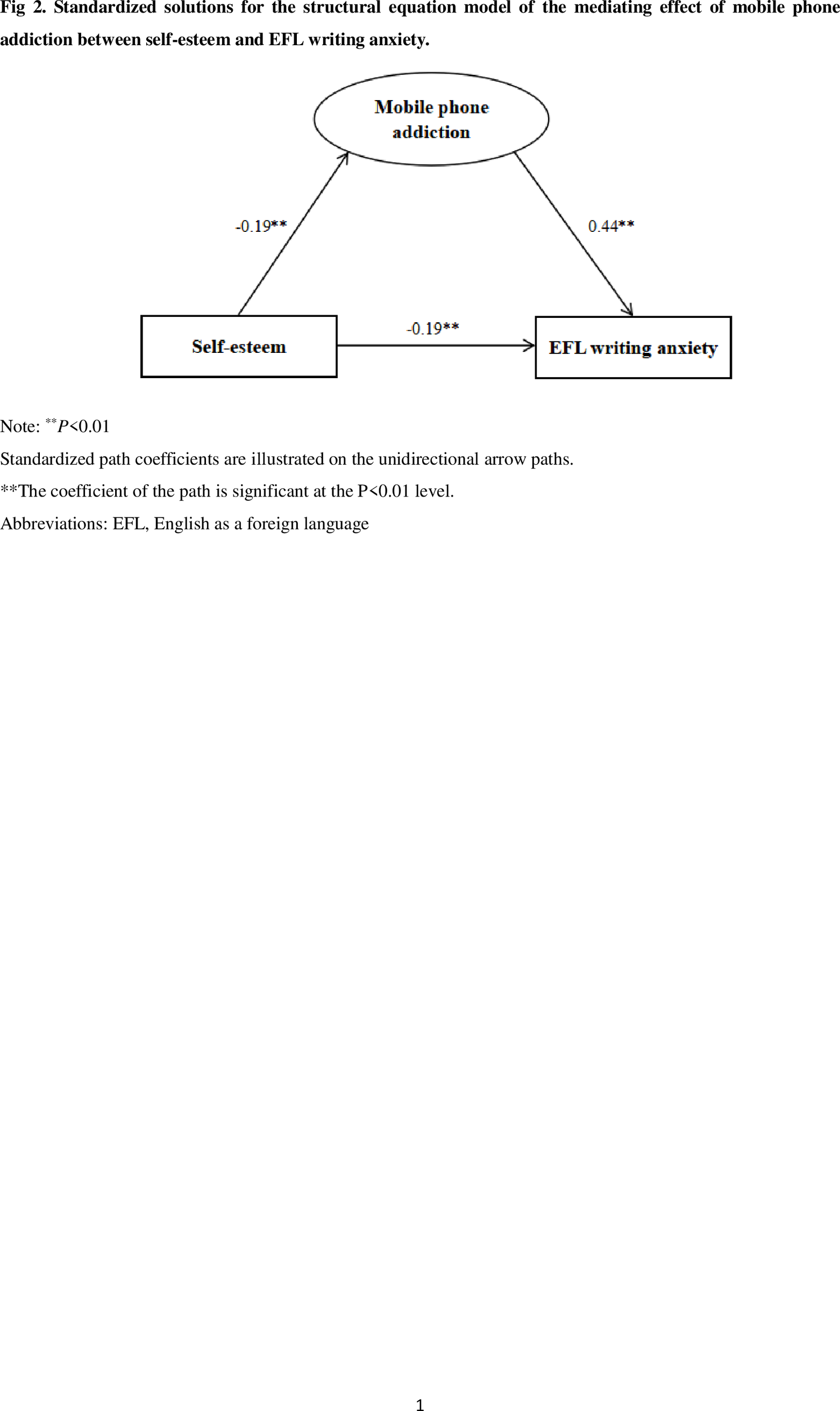

Supplement: S2 Fig — (TIF) [file pone.0284335.s002.tif]

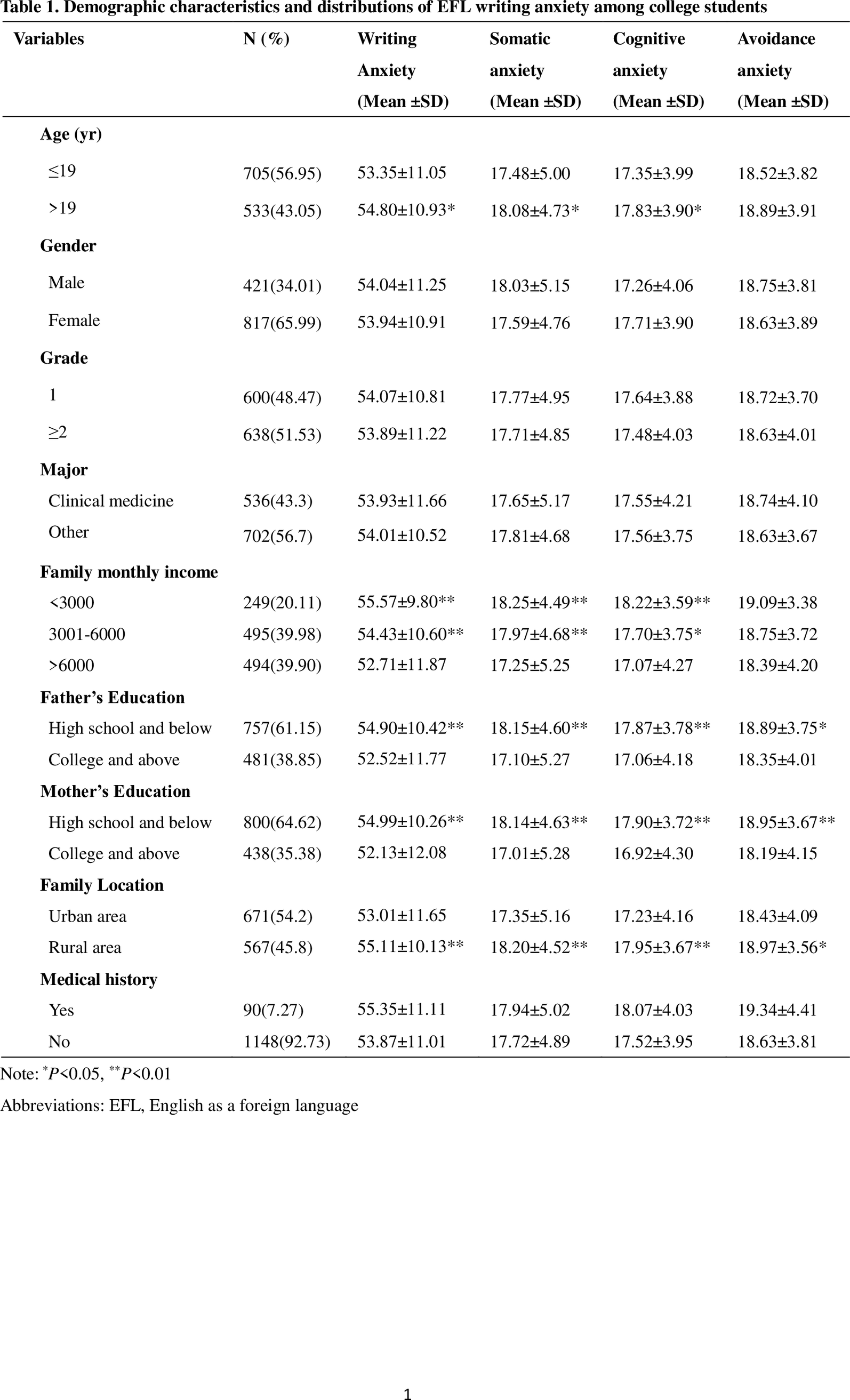

Supplement: S1 Table — (TIF) [file pone.0284335.s003.tif]

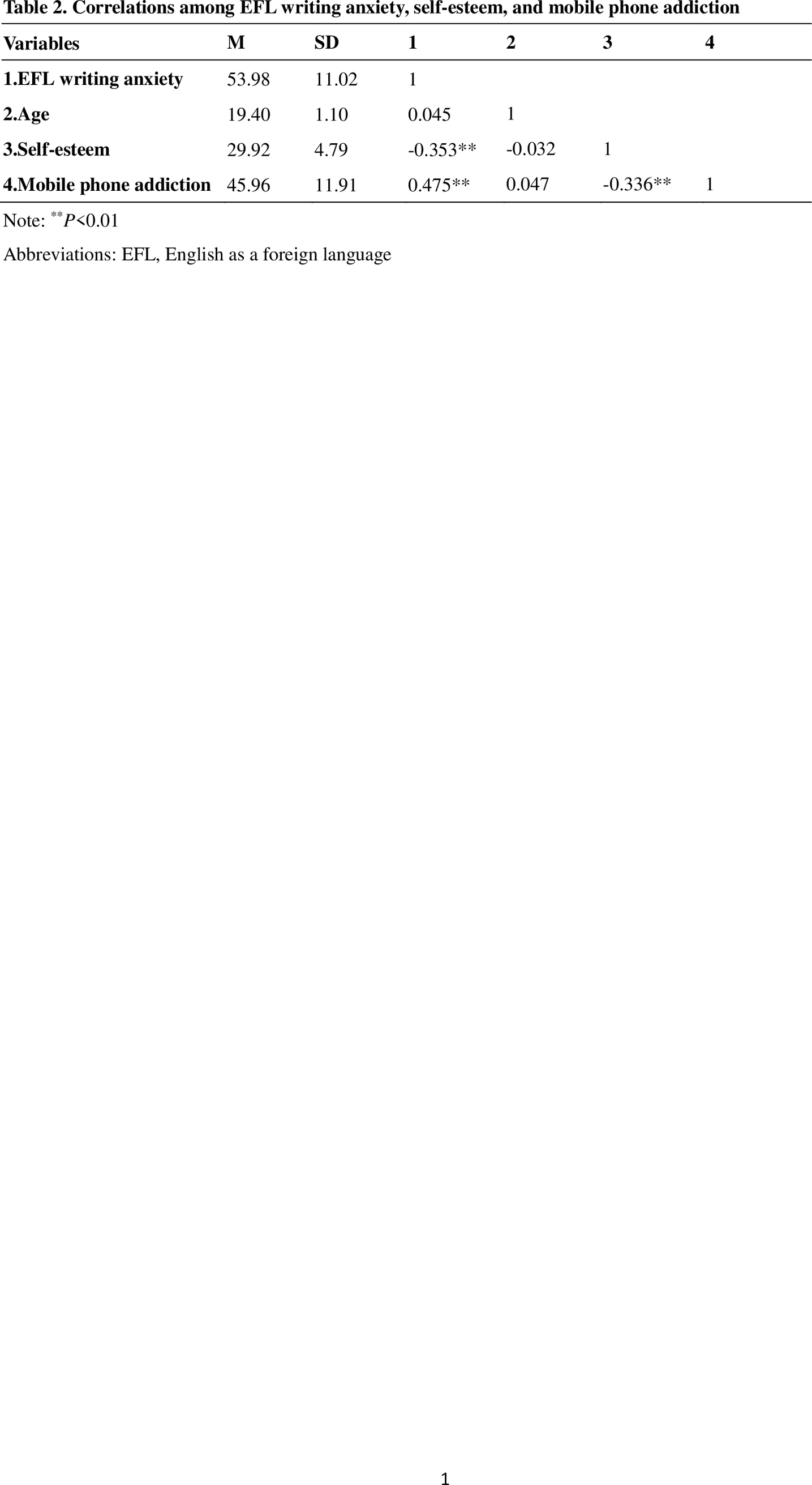

Supplement: S2 Table — (TIF) [file pone.0284335.s004.tif]

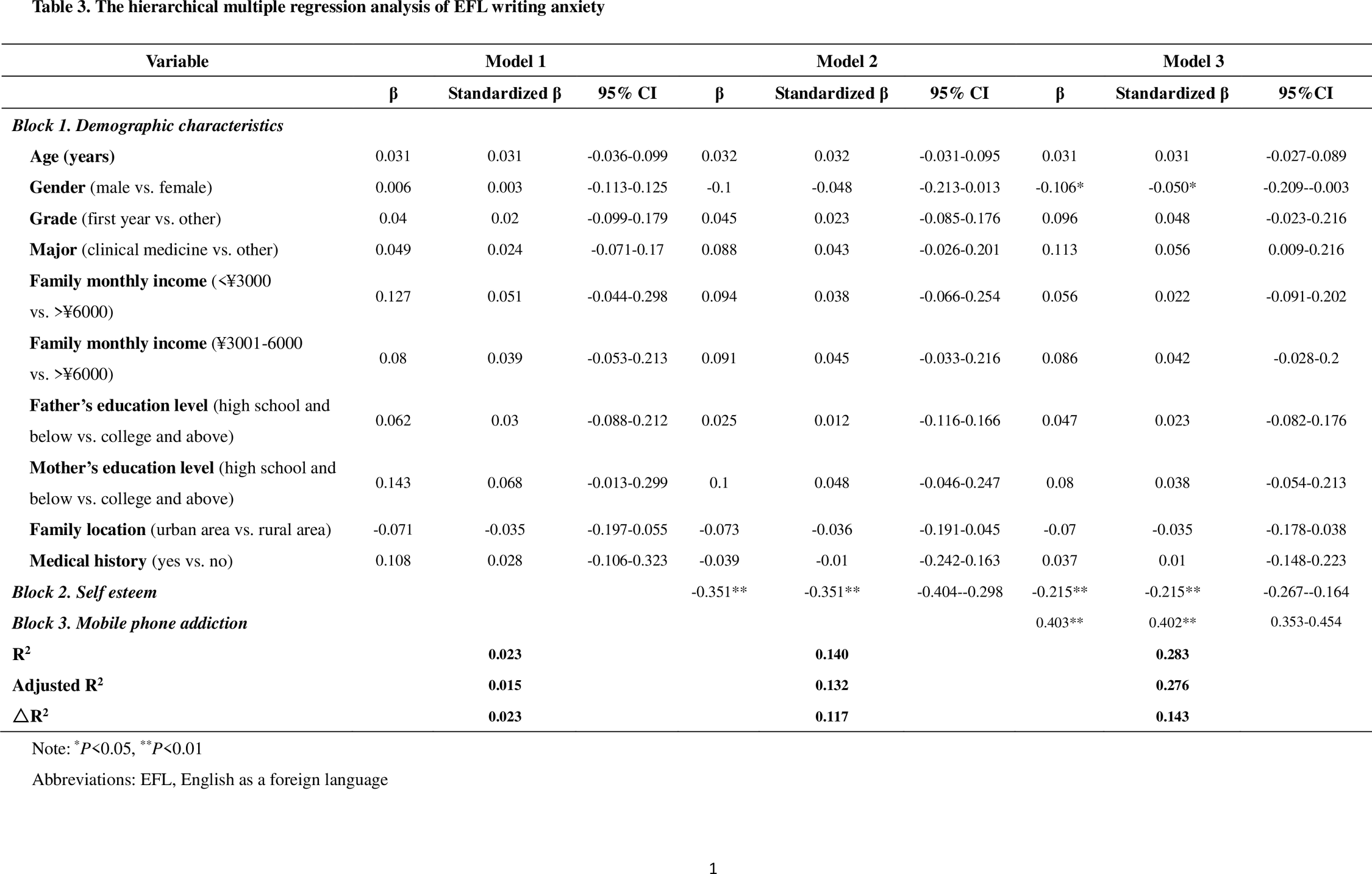

Supplement: S3 Table — (TIF) [file pone.0284335.s005.tif]

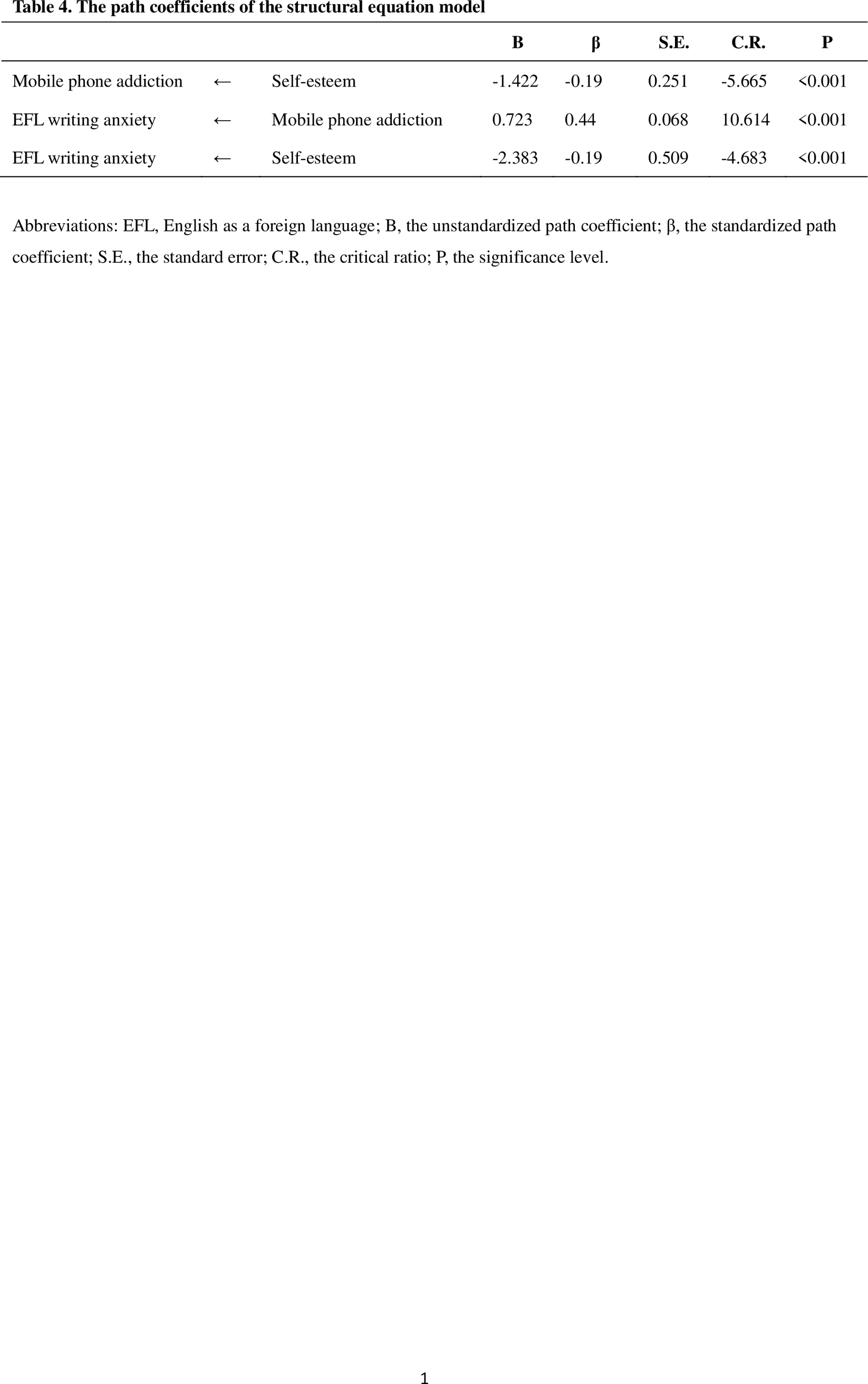

Supplement: S4 Table — (TIF) [file pone.0284335.s006.tif]
